# Recruitment and Retention Strategies in Clinical Trials for Hip Fractures: A Cross-Sectional Analysis

**DOI:** 10.5435/JAAOSGlobal-D-24-00242

**Published:** 2025-04-29

**Authors:** Josh Major, Kurt Mahnken, Merhawit Ghebrehiwet, Payton Clark, Josh Autaubo, Andrew Wilson, Jake Checketts, Alicia Ford, Matt Vassar

**Affiliations:** From the Office of Medical Student Research, Oklahoma State University Center for Health Sciences, Tulsa, OK (Mr. Major, Mr. Mahnken, Ms. Ghebrehiwet, Ms. Clark, Mr. Autaubo, Mr. Wilson, Dr. Ford, and Dr. Vassar); the Department of Orthopaedic Surgery, Oklahoma State University Medical Center, Tulsa, OK (Mr./Dr. Checketts); and the Department of Psychiatry and Behavioral Sciences, Oklahoma State University Center for Health Sciences, Tulsa, OK (Dr. Ford and Dr. Vassar).

## Abstract

**Objective:**

To investigate the recruitment and retention strategies in clinical trials evaluating hip fractures, with a focus on underrepresented populations.

**Background:**

The prevalence and burden of hip fractures necessitate diverse and representative clinical trials to improve management outcomes. Underrepresented populations often face barriers to participation, affecting the generalizability of trial results.

**Methods:**

We conducted a cross-sectional analysis of clinical trials on hip fractures published between 2018 and 2023. We searched Embase and MEDLINE, screening and extracting data in a masked duplicate manner. For statistical analysis, Stata 18 SE (StataCorp LLC) was used to determine frequencies of recruitment and retention strategies.

**Results:**

We screened 624 studies, with 72 meeting our inclusion criteria. Trials were conducted in the United States (12/72, 16.7%), non-United States (53/72, 73.6%), or both (7/72, 9.7%). Only one trial (1.4%) mentioned specific recruitment strategies, and three (4.2%) reported measures to minimize participant dropout. Overall, the mention of strategies for diverse participation was scarce.

**Conclusion:**

There is a notable lack of recruitment and retention strategies directed at promoting diverse participation in hip fracture trials. This study highlights the need for improved inclusiveness and equity in future clinical trials to enhance the generalizability of their findings and better serve all populations affected by hip fractures.

Hip fractures are a notable health concern globally among aging populations, with a substantial effect on morbidity, mortality, and healthcare costs. Furthermore, the global burden of disease surrounding hip fractures and their associated morbidities is steadily increasing.^1^ In the United States alone, approximately 300,000 hip fractures occur annually. This number is projected to increase as the population ages.^1^ Even following surgical fixation, patients may incur lasting effects from fracturing their hip, including decreased mobility and loss of independence.^2^ The management of hip fractures poses a notable challenge to healthcare systems, as they are often associated with lengthy stays and other associated logistical challenges.^3^ These facts highlight the importance of effective treatment strategies and interventions to improve outcomes for people with hip fractures.

Despite advancements in technology and perioperative care, inequities persist in the incidence, management, and outcomes of hip fractures among different demographic groups.^4^ Some populations, including people from racial and ethnic minority groups, persons from lower socioeconomic backgrounds, and those residing in rural areas, experience a disproportionately higher burden of hip fractures.^2^ Furthermore, Amen et al^4^ brought to light the poor outcomes that are especially prevalent for Black patients. Although Amen et al focused on hip fracture outcomes in the United States, international studies highlight similar inequities. For example, research conducted in the United Kingdom by Nisar et al^5^ found that South Asian and Black populations experienced higher rates of delayed surgical intervention and worse postoperative outcomes compared with White patients. Similarly, a systematic review of hip fracture management in China identified sociodemographic disparities in access to postoperative care, leading to poorer health-related quality of life outcomes for disadvantaged populations.^6^ These findings underscore the global nature of inequities in hip fracture management and the need for diverse and inclusive clinical trials to address these inequities comprehensively.

Clinical trials play a crucial role in evaluating the efficacy and safety of interventions.^7^ However, the generalizability of clinical trials to diverse patient populations relies on the recruitment and retention of participants from these populations. Poor inclusion of diverse populations can severely limit the ability to draw meaningful conclusions about the effectiveness of interventions in patients not represented in the included data. Given the high incidence of hip fractures, it is crucial to understand the strategies used to recruit and retain participants from diverse populations in clinical trials. These trials inform the management of this frequently encountered condition, and their generalizability is essential to ensuring that clinical guidelines are applicable across diverse patient groups.^8^

A lack of diversity in clinical trials can result in efficacy and safety profiles that do not reflect the experiences of all patient groups. For instance, differences in pharmacokinetics and pharmacodynamics among racial and ethnic groups may result in varying drug metabolism and responses, as evidenced in studies involving anticoagulants and antihypertensive medications.^9^ Similarly, inequities in access to care, cultural differences, and comorbid conditions may exacerbate health inequities when trial outcomes are used to guide clinical practice.^10^ Thus, the absence of diversity can inadvertently perpetuate inequities in treatment outcomes, particularly for underrepresented populations, such as racial minorities, rural residents, and those with lower socioeconomic status.^10^

This study aims to examine the recruitment and retention strategies employed in clinical trials focusing on hip fractures and their effect on the generalizability of study findings. By identifying potential inefficiencies and barriers to recruitment and retention, this study seeks to improve the design and implementation of future clinical trials regarding the treatment and management of hip fractures. Ultimately, this research aims to enhance our understanding of how to effectively recruit and retain participants from diverse populations in clinical trials for hip fractures, leading to more inclusive and representative study populations and, ultimately, improved outcomes for all people affected by this debilitating condition.

## Methods

### Study Design

In this cross-sectional study, we investigated the use of recruitment and retention strategies in clinical trials involving hip fractures. We followed relevant guidelines from the Preferred Reporting Items of Systematic Reviews and Meta-Analyses.^11,12^ The guidelines Strengthening the Reporting of Observational Studies in Epidemiology were not adhered to as we employed systematic review methods. Our study was submitted for institutional review board approval; they determined that this study did not meet the definition of human subjects research according to the United States Code of Federal Regulations.^13^ To promote reproducibility and transparency, our study protocol, search strategy, data, pilot-tested Google Forms, and statistical analyses were uploaded a priori to Open Science Framework.^14^ This study was conducted alongside other studies with a similar methodology.

### Use of Language

The language used in this article adheres to standardized terminology from *Advancing Health Equity: A Guide to Language, Narrative, and Concepts* by the American Medical Association.^15^

### Search Strategy

We searched the Cochrane Database of Systematic Reviews for published systematic reviews on hip fractures. These reviews were examined to identify relevant terms and keywords related to our topic. PubMed was then used to determine the most appropriate tags for each keyword. Medical Subject Headings terms (i.e., Hip Fractures [Medical Subject Headings Terms]) were employed for the development of our search string ensuring a comprehensive approach. On May 28, 2024, we searched Embase (Elsevier) and MEDLINE (PubMed) to identify clinical trials involving hip fracture and its associated treatments and interventions. Search returns were subsequently uploaded to Rayyan (http://rayyan.qcri.org/). Two investigators (J.M., K.M.) screened titles and abstracts in a masked, duplicate manner, ensuring objectivity and adherence to practice guidelines outlined by Cochrane.^16^

### Eligibility Criteria

The clinical trials included in our study met the following criteria: (1) publication date between January 1, 2018, and December 31, 2023, (2) analyzed an intervention—therapeutic, behavioral, supplemental/holistic, or other—and its associated outcomes on participants within the study, (3) enrolled patients with a fractured hip or focused on the prevention of fracture in patients with increased risk, and (4) conducted at national or multinational trial sites with an Ethnic Fractionalization Index (EFI) ≥ 0.3. The EFI measures the ethnic diversity of a country and represents the degree of heterogeneity within a population.^17^ However, it does not directly account for socioeconomic diversity, which is often closely intertwined with racial and ethnic factors. Although racial diversity is a critical aspect of inclusivity, it is important to recognize that socioeconomic inequalities may act as confounding factors, influencing access to health care and trial participation independently of race.^18^ For our study, if a clinical trial was conducted at multiple sites internationally, we calculated the mean EFI using the EFI scores from each location. If the number of study participants at each individual site was available, a weighted EFI was calculated. To ensure countries that lacked diverse populations were not unjustly analyzed, an EFI below 0.3 resulted in exclusion from our study.

We excluded studies that were (1) secondary analyses, (2) interim analyses, (3) erratum, (4) corrigendum, and (5) trial updates. The two authors responsible for screening (J.M., K.M.) reconciled findings, with a third author (M.G.) available to solve any disputes.

### Data Extraction

Two authors (J.M., K.M.) extracted in a masked, duplicate fashion using a pilot-tested Google Form. The following general data characteristics were recorded: first author name, corresponding author name and e-mail address, total number of authors, author first names, publication year, sources of funding, intervention classification (eg, therapeutic, behavioral, supplement/holistic, other), desired population (generally by disease, specific disease subgroups, and comorbidities of the diseases), study location, participant age (mean and range), efficacy outcomes (classified as clinical outcomes, clinical scales, or surrogate outcomes), study duration, use of randomization, use of masking (none, single, double or greater), intention-to-treat sample size, number of participants lost to follow-up. Although data on whether studies reported participant race/ethnicity and sex assigned at birth/sex identity were collected, these variables were not analyzed in detail. This decision was made to maintain the focus of this article on recruitment and retention strategies in clinical trials rather than on participant diversity itself. However, our recorded findings are available on Open Science Framework.^14^ Future studies could address the reporting and implications of demographic characteristics as a separate area of inquiry.

We extracted any strategies mentioned that were directed at recruiting or retaining participants, especially for historically underrepresented populations. We recorded the following retention-related strategies: measures taken to limit participant dropout, execution of planned diversity goals, and ethical considerations. The methods and limitations sections were analyzed for any discussion about recruitment-related challenges or successful strategies.

### Statistical Analysis

General study characteristics and the use of recruitment and retention strategies were presented in frequencies and percentages. Data analyses were conducted using Stata 18 SE (StataCorp LLC).

Furthermore, we computed either an unweighted or weighted mean EFI for trials that involved multiple countries. This decision was based on whether the trial provided detailed sample sizes of the participating countries or not. For the unweighted mean EFI (where such information was not provided), we calculated an unweighted mean of the EFIs of each participating country. For the weighted mean EFI (when sample sizes for each country were reported), we multiplied the number of participants from each country by that country's EFI. We then summed these products and divided the total by the overall number of participants. The EFIs for the included studies can be found in the Supplementary Index (http://links.lww.com/JG9/A405).

## Results

### Trials Inclusion and Exclusion

**Figure 1:**
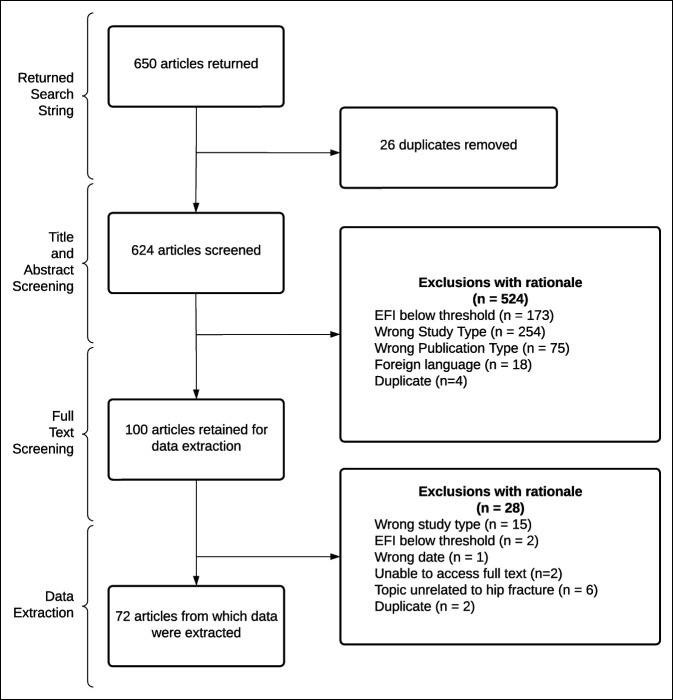
Flow diagram displaying exclusion rationales throughout the screening and data extraction processes. PRISMA = Preferred Reporting Items for Systematic reviews and Meta-Analyses

Our search returned 650 results. After removing duplicates, 624 articles were screened to determine whether the inclusion criteria were met. Following exclusions, 100 articles remained for the data extraction; an additional 28 articles were excluded upon full-text screening. Detailed descriptions of exclusions with rationale are available in Figure 1.

### Trial Characteristics

Seventy-two clinical trials met our inclusion criteria. Most these trials (53/72, 73.6%) took place at non–United States sites. The remaining either took place in the US (12/72, 16.7%) or a combination of US and international sites (7/72, 9.7%). Trial participants were randomized in 70 (97.2%) of the 72 included trials. None of the studies in our sample employed greater than double-blinded masking, whereas most trials reported no blinding or made no mention of it (31/72, 43.1%). Most studies involved therapeutic interventions (60/72, 83.3%). Table 1 provides detailed trial characteristics.

**Table 1:** General Characteristics of Included Studies (*n = 72*)

General Characteristics	Frequency (%)
Time study was conducted	
2018	8 (11.1)
2019	13 (18.1)
2020	12 (16.7)
2021	13 (18.1)
2022	10 (13.9)
2023	16 (22.2)
Location	
United States of America	12 (16.7)
International, including United States	7 (9.7)
International, excluding United States	53 (73.6)
Randomization	
Yes	70 (97.2)
No	2 (2.8)
Masking	
Single	20 (27.8)
Double	21 (29.2)
Greater than double	0 (0.0)
None	31 (43.1)
Funding	
Private	10 (13.9)
Industry	6 (8.3)
Self-funded	0 (0.0)
Hospital/university	4 (5.6)
Government	9 (12.5)
No funding	16 (22.2)
Not mentioned	15 (20.8)
Mixed	12 (16.7)
Classification of intervention	
Therapeutic	60 (83.3)
Behavioral	9 (12.5)
Supplemental/holistic	3 (4.2)
Other	0 (0.0)

### Recruitment and Retention Strategies

Of the clinical trials in our study, only one trial (1.4%) mentioned specific recruitment strategies for obtaining their participants (Table 2). That study recruited participants through fliers, e-mail announcements, information sessions, and advertisements. Planned diversity goals were seldom mentioned, with only 4 (5.6%) of the 72 studies including such statements. Similarly, very few studies mentioned ethical considerations concerning recruiting a diverse population (2/72, 2.8%). Only three studies implemented measures to minimize participant dropout (3/72, 4.2%). Some examples of retention methods contained in these studies are financial compensation for follow-up visits and the exclusion of participants who were deemed likely to present problems with follow-up.

**Table 2:** Summary Table for Recruitment and Retention Strategy Usage

Item Assessed	Frequency (%)	
	Yes	No
1. Did the study mention recruitment strategies?	1 (1.4)	71 (98.6)
2. Was a planned diversity goal mentioned in the methods?	4 (5.6)	68 (94.4)
3. Were any considerations/ethical approvals taken concerning diversity recruitment?	2 (2.8)	70 (97.2)
4. Are any limitations/challenges to recruitment mentioned?	11 (15.3)	61 (84.7)
5. Were any measures taken to prevent participant dropout?	3 (4.2)	69 (95.8)

Only a small portion of the studies discussed any limitations or challenges related to recruitment (11/72, 15.3%). The most common of these limitations involved the exclusion of participants who did not speak the language of the region where the trial took place. Table 2 includes information regarding these strategies and their usage.

## Discussion

The results of our study reveal a widespread lack of recruitment and retention strategies aimed at promoting participant diversity in clinical trials on hip fractures. Many of these trials experienced high loss-to-follow-up rates, primarily because of the absence of tactics directed toward participant retention. In addition, very few studies had set diversity goals or took steps to ensure the inclusion of diverse populations. This lack of diversity predominantly affects historically underrepresented populations, resulting in the diminished generalizability of the trials' outcomes. A recurrent limitation was the exclusion of participants who did not adequately speak or understand the language of the trial region, further reducing the diversity of study populations. The overarching theme among hip fracture trials is the insufficient attention to diverse recruitment and retention, leading to poor representation of historically excluded communities. The implications of poor inclusion are multifaceted. When underrepresented groups are excluded, trial data may fail to account for variations in treatment response because of genetic, environmental, and sociocultural factors. For example, certain interventions that demonstrate efficacy in homogeneous populations may not translate to broader demographics, leading to suboptimal care.^19^ A lack of inclusivity also means that potential adverse effects specific to underrepresented populations may go undetected, compromising patient safety. Moreover, healthcare guidelines based on nondiverse trials may inadvertently reinforce existing disparities, as clinicians apply findings to populations that were not adequately studied.^20^ Consequently, it is imperative to enhance recruitment and representation strategies in clinical trials to address inequities in treatment and outcomes for patients from diverse populations.

Our findings align with the existing literature that highlights the deficiencies in recruitment and retention efforts for historically underrepresented populations in clinical trials. Barriers such as language differences, literacy levels, family composition, demographics, symptom severity, and historical mistrust in medical research markedly impede the participation of people from minority groups in clinical trials.^21^ Similarly, our study found that very few trials (5.6%) had planned diversity goals, and only 2.8% took specific steps to diversify their recruitment strategies. Certain exclusion criteria, such as those excluding patients with comorbidities, disproportionately affect people from certain minority groups, further reducing their participation in clinical trials.^19^ These findings underscore the need for methodological changes and policy interventions to enhance the inclusion of diverse populations in clinical research.

Our findings have concerning implications. Orthopaedic surgery presents notable difficulties in creating clinical trials of high quality, as the surgical interventions make randomization or prospective data collection challenging in many ways. Because of this, the scarcely produced clinical trials are frequently included in practice guiding documents (such as clinical practice guidelines, appropriate use criteria, and the like) that serve as standard of care benchmarks. Furthermore, the trials and subsequent practice guiding documents serve as data sources for clinical practice aides (such as Up To Date) and also as references for healthcare policy and reimbursement committees. If the trials providing the foundation for evidence-based orthopaedic surgery have poor recruitment and retention strategies for certain populations, it is easy to imagine the negative downstream effects this lack of generalizability will have on clinical practice.

To address the current inadequacies in recruitment and retention strategies in hip fracture trials, several improvements in trial design and logistics can be made. Providing interpreters to overcome language barriers would help in obtaining consent and delivering study materials in participants' native languages. Engaging local community members in the recruitment and onboarding process can build trust and increase community participation.^22^ Evidence from prior studies supports the efficacy of these strategies. For example, the Jackson Heart Study, which focused on cardiovascular disease among African Americans, successfully enhanced recruitment and retention through extensive community engagement, including partnerships with local organizations, culturally tailored recruitment materials, and the inclusion of community advisory boards.^23^ Similarly, the Hispanic Community Health Study/Study of Latinos used bilingual staff, culturally appropriate materials, and extended outreach efforts to address language barriers and foster trust. These approaches led to robust participation rates, demonstrating the feasibility of overcoming logistical and sociocultural barriers through targeted strategies.^24^ Selecting study sites that are easily accessible to diverse populations, including outpatient clinics, can enhance participant diversity. Educating participants and their caregivers about the significance of their participation in trials may also improve retention. Formulating and adhering to a predefined framework for diversifying study populations during trial planning and execution is crucial.^25^ In addition, leveraging technology to reach historically underrepresented communities can help increase their participation in clinical trials.^26,27^ Many of these strategies have been effectively used by the national institutes of health (NIH's) All of Us Research Program. This program leverages digital tools such as mobile applications and social media to reach historically excluded communities.^28^ By implementing flexible enrollment processes, offering financial compensation for time and travel, and conducting surveys in multiple languages, the initiative has successfully recruited participants from rural areas, ethnic minorities, and other underrepresented groups.^29^ These success stories highlight the transformative potential of combining technology, community engagement, and cultural competency to address inequities in clinical trial participation.

We also provide the following research-directed recommendations to address the evident gaps in recruitment and retention strategies for historically underrepresented populations in hip fracture clinical trials. First, to increase awareness and the relevance of recruiting and retaining historically underrepresented populations in hip fracture clinical trials, we recommend that journal instructions for authors include a statement recommending an included statement regarding how they ensured the population of their study was generalizable. Previous work has demonstrated increased adherence and reporting when journal policies require or recommend methodological safeguards.^30^ This statement could be required similar to funding and conflict of interest statements and should follow the guidelines set by the New England Journal of Medicine, which requires a supplementary table that provides background information on the disease, problem, or condition and the representativeness of the study group, to be posted with the article at the time of publication.^31^ We also recommend that leaders involved in developing reporting guidelines discuss the benefits of including diversity measures in commonly used guidelines, such as CONSORT. At present time, the most commonly used reporting guidelines do not mention reporting measures directed toward the recruitment and retention of diverse populations. We also recommend funding agencies like the NIH prioritize and allocate grants to studies that have well-defined diversity goals and demonstrate a commitment to recruiting participants from underrepresented communities. The NIH Revitalization Act of 1993 mandates the inclusion of women and patients in clinical research; however, more stringent enforcement and regular audits are necessary to ensure compliance.^32^ Regulatory bodies should also require transparent reporting on the demographics of trial participants and the strategies employed to achieve diversity, as highlighted by the Food and Drug Administration's guidance on enhancing diversity in clinical trials.^33^ Implementing these policy changes will foster a more inclusive research environment, leading to more generalizable and applicable study outcomes that better serve all populations affected by hip fractures.

Our study has several strengths. First, it adhered to the Preferred Reporting Items of Systematic Reviews and Meta-Analyses guidelines, ensuring a systematic and transparent approach to data collection and analysis, which enhances the reliability and reproducibility of our findings.^6^ Second, our cross-sectional analysis included a diverse range of clinical trials from multiple countries, providing a comprehensive overview of current recruitment and retention strategies in hip fracture research. This international scope allows for broader generalizability of our findings. Third, the use of masked duplicate screening and data extraction minimized potential bias and increased the accuracy of our results.

However, our study also has limitations. One major limitation is the reliance on published data, which may be subject to reporting biases. Studies that failed to report specific recruitment and retention strategies or challenges may not be accurately represented in our analysis.^34^ In addition, our study was limited to trials published between 2018 and 2023, which may not capture longer term trends in recruitment and retention practices. The exclusion of studies conducted in countries with an EFI below 0.3 may also limit the generalizability of our findings to less diverse populations. Finally, although our analysis included a range of interventions and settings, the specific contextual factors influencing recruitment and retention in each trial were not always fully detailed, which could affect the interpretation of our results.

## Conclusion

Our study underscores notable deficiencies in the recruitment and retention strategies of clinical trials for hip fractures, particularly regarding the inclusion of historically excluded populations. The lack of diversity plans and strategies undermines the generalizability and validity of trial outcomes, perpetuating health inequities. To address these gaps, comprehensive policies and practical strategies, such as community engagement, culturally and linguistically appropriate resources, and enhanced researcher training, are essential. Funding agencies and regulatory bodies must enforce stricter guidelines and support diverse recruitment efforts. By prioritizing inclusivity, future research can become more representative and impactful, leading to better health outcomes for all populations affected by hip fractures. Continued efforts and innovations are necessary to bridge the gap between research and real-world patient populations, ensuring equitable healthcare outcomes.
